# Which is the optimal choice for neonates’ formula or breast milk?

**DOI:** 10.1007/s13659-024-00444-0

**Published:** 2024-03-15

**Authors:** Yueqi Hu, Xing Wu, Li Zhou, Jikai Liu

**Affiliations:** National Demonstration Center for Experimental Ethnopharmacology Education, School of Pharmaceutical Sciences, South-Central MinZu University, Wuhan, 430074 People’s Republic of China

**Keywords:** Human milk, Physico-chemical properties, Human milk bioactive, Phospholipids, Mammalian milk

## Abstract

The incidence of prematurity has been increasing since the twenty-first century. Premature neonates are extremely vulnerable and require a rich supply of nutrients, including carbohydrates, proteins, docosahexaenoic acid (DHA), arachidonic acid (ARA) and others. Typical breast milk serves as the primary source for infants under six months old to provide these nutrients. However, depending on the individual needs of preterm infants, a more diverse and intricate range of nutrients may be necessary. This paper provides a comprehensive review of the current research progress on the physical and chemical properties, biological activity, function, and structure of breast milk, as well as explores the relationship between the main components of milk globular membrane and infant growth. Additionally, compare the nutritional composition of milk from different mammals and newborn milk powder, providing a comprehensive understanding of the differences in milk composition and detailed reference for meeting daily nutritional needs during lactation.

**Graphical Abstract:**

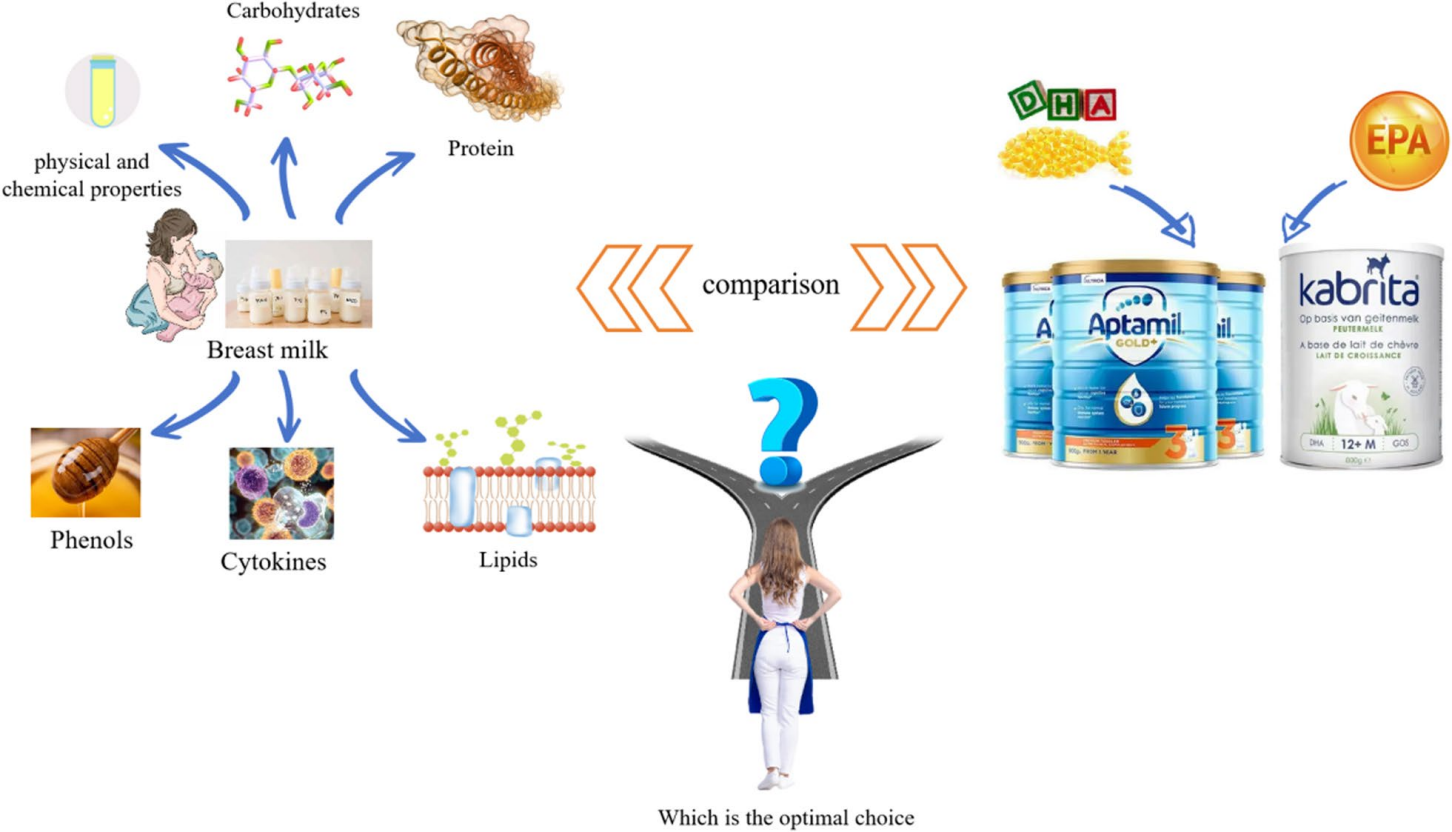

## Introduction

Breast milk analysis, as an extensively researched field, serves as the primary source of nutrition and bioactive compounds for neonates. It encompasses five main components: carbohydrates (including lactose and human milk oligosaccharides (HMOs) [1], proteins (such as lactoferrin, lysozyme, secretory IgA (sIgA), haptocorrin, α-lactalbumin, bile salt-stimulated lipase, k-casein, β-casein), lipids (including phospholipids found in milk fat globule membrane (MFGM) chitin), phenolics, and cytokines. HMOs exhibit antimicrobial activity that effectively protects infants against harmful pathogens [2]. Lactose serves as an energy source for growth and contributes to weight gain in infants [3]. Proteins are the primary nutrients in breast milk and provide essential bioactive substances crucial for infant growth and development [4]. Lipids play a vital role by providing energy for growth and development while also supporting neurological and brain development during infancy [5]. A strong correlation has been observed between the cytokines and growth factors present in breast milk and the onset of specific infant ailments [6]. Breast milk is widely recognized by the medical community as the optimal source of nutrition for infants. In cases where a mother’s own milk is unavailable or insufficient, such as with vulnerable preterm infants, donor human milk from breast milk banks can serve as a valuable substitute to significantly reduce morbidity and mortality rates [7]. DHA and ARA are widely recognized as crucial nutrients in breast milk, with active lipid molecules being the most prevalent additives found in currently available infant formulas on the market. Consequently, they receive considerable attention from mothers when selecting a formula for their infants. However, many mothers may not fully comprehend the practical applications or specific roles of these two substances beyond their importance to infant development. In fact, from a scientific perspective, the carbohydrates, proteins, phenolic compounds, cytokines and lipids (including DHA and ARA) present in breast milk all exhibit corresponding physiological activities. Specifically regarding DHA and ARA, the primary focus of their effects lies within neural growth within the brain [8]. Furthermore, in comparison to those who received normal amounts, it has been demonstrated that infants fed low levels of DHA and ARA exhibited elevated levels of omega-6 fatty acids. However, studies have also shown that omega-6 may hinder the growth of secondary neurotransmitters and reduce docosahexaenoic acid in the developing brain [9]. An increasing body of study exploring the correlation between breast milk functionality and infant development. For instance, during the recent COVID-19 outbreak, significant IgA-dominant immune responses against SARS-CoV-2 were detected in the breast milk of convalescent mothers [10, 11], presenting a novel approach and idea for treating emerging coronaviruses by isolating antibody-active material from recovered mothers’ breast milk [12]. Breast milk is not only effectively treating novel coronaviruses, but also provides infants with essential immunity, active nutrients and probiotics to promote growth and safeguard intestinal flora. As evidenced by reports from the United Nations and some studies on neonatal deaths caused by nutritional problems annually, breast milk is closely linked to infant health. Furthermore, some research has shown that non-breastfed infants have a higher risk of major diseases compared to those who are breastfed [13, 14]. This paper presents a comparative and comprehensive analysis of the composition and content of key substances in breast milk under various conditions, while also providing a horizontal comparison with the milk of different mammalian species to visually illustrate their distinctive characteristics. The findings aim to offer valuable insights and references for promoting optimal growth, development, and early nutrient intake in infants.

## Main composition and properties of milk

### physical and chemical properties

The acidity of breast milk is primarily influenced by casein, minerals organic acids, carbon dioxide and citrate. However, the pH value of milk may decrease to varying degrees over time and during storage, which can serve as an indicator for potential quality issues with the milk [15]. Fresh breast milk usually has a pH value of around 7 [16]. Mature breast milk samples frozen at − 20 °C for three months exhibited significantly lower pH and significantly higher titratable acidity values compared to fresh breast milk (Table 1) [17, 18]. The main factors contributing to these changes in pH are the hydrolysis of milk lipids and proliferation of bacterial populations. Over time under basic environmental conditions, biological enzymes and bacteria break down lipids in the milk into free fatty acids, resulting in a decrease in pH. However, experimental data has demonstrated the presence of pH fluctuations in milk, which can be attributed to its inherent buffering capacity. The primary buffering compounds in milk include soluble calcium phosphate salts, citric acid, bicarbonate, as well as the acidic and basic amino acid side chains found on proteins, particularly casein [19–21].

**Table 1:** Physicochemical parameters of the fresh human milk (*) and frozen samples in different periods of lactation (x ± SD) [17, 18]

Period of lactation	Electrical conductivity (mS/cm)	Refractive index	Dynamic viscosity (Pas 10^**–3**^)	Surface tension (10^**–**3^ N/m)	Titratable acidity (% lactic acid)	pH
4th day	1.89 ± 0.02	1.35 ± 0.00	2.40 ± 0.01	47.73 ± 1.50	0.070 ± 0.004	6.99 ± 0.03
10th day	1.55 ± 0.03	1.35 ± 0.00	1.44 ± 0.01	47.66 ± 1.10	0.051 ± 0.005	7.07 ± 0.03
20th day	1.46 ± 0.02	1.35 ± 0.00	1.46 ± 0.01	45.60 ± 1.35	0.023 ± 0.008	7.24 ± 0.05
30th day	1.36 ± 0.03	1.35 ± 0.00	1.39 ± 0.01	44.91 ± 1.10	0.075 ± 0.004	6.36 ± 0.01
6th week	1.30 ± 0.02	1.35 ± 0.00	1.35 ± 0.01	40.81 ± 0.95	0.090 ± 0.005	6.36 ± 0.02
9th week	1.40 ± 0.02	1.34 ± 0.00	1.40 ± 0.01	37.14 ± 0.70	0.085 ± 0.005	6.26 ± 0.01
10th week	1.33 ± 0.03	1.34 ± 0.00	1.35 ± 0.01	42.51 ± 0.95	0.105 ± 0.007	6.26 ± 0.04
12th week	1.42 ± 0.03	1.3 ± 0.00	1.28 ± 0.01	35.96 ± 0.80	0.100 ± 0.003	6.34 ± 0.03
14th week	1.43 ± 0.02	1.34 ± 0.00	1.40 ± 0.01	36.97 ± 0.85	0.108 ± 0.005	6.20 ± 0.05
4th month	1.38 ± 0.04	1.35 ± 0.00	1.39 ± 0.01	37.02 ± 0.95	0.125 ± 0.004	6.13 ± 0.04
5th month	1.36 ± 0.04	1.35 ± 0.00	1.32 ± 0.01	34.62 ± 0.75	0.098 ± 0.005	6.25 ± 0.04

The color of breast milk is also one of its most important physical and chemical properties, can also reflect the composition and physiological status problem of certain chemicals. The white color of milk primarily arises from the scattering of light by casein micelles (CMs) and milk fat globules (MFG), while variations in CM size contribute to the color changes observed in skimmed milk [21]. Notably, there have been reports of a moderately preterm mother with green-colored breast milk, despite her adherence to a proper diet and medication regimen. Intriguingly, her newborn appeared healthy without any apparent issues, and over time the green hue in her breast milk dissipated. Although ample evidence suggests that maternal food choices or medications can influence the coloration of breast milk, research findings specifically addressing food and medication-induced discoloration remain scarce [22]. In addition, there was a temporary occurrence of purple breast milk, leading to a precautionary pause in breastfeeding, however, the color returned to its normal state within a week. Subsequently, ultrasound examinations revealed slight ductal protrusion in both breasts. The surgeon attributed this staining to ductal dilation and hormonal stimulation and confirmed no adverse effects on the infant from the presence of purple breast milk [23]. Generally speaking, variations in the color of breast milk can occur. For instance, green hue may be associated with the consumption of vegetables or beverages containing green additives and potentially influenced by isoproterenol. Rusty tube syndrome represents a pathological phenomenon where blood and degradation products pass through lactation ducts resulting in slightly red, green or brown colored breast milk [24]. Nevertheless, these colors are essentially harmless and transient. Therefore, it is crucial to appropriately recognize such occurrences during breastfeeding.

### Carbohydrates

Lactose, a key constituent of breast milk, serves as a vital energy source for infants during the early stages of development. Its content remains relatively stable throughout lactation without significant fluctuations [25]. Apart from providing essential energy, lactose plays a crucial role in regulating milk osmotic pressure and enhancing the absorption of critical minerals like calcium [26]. This phenomenon may be attributed to the conversion of lactose into lactic acid within the digestive tract, leading to pH reduction and alterations in mineral solubility.

**Fig. 1:**
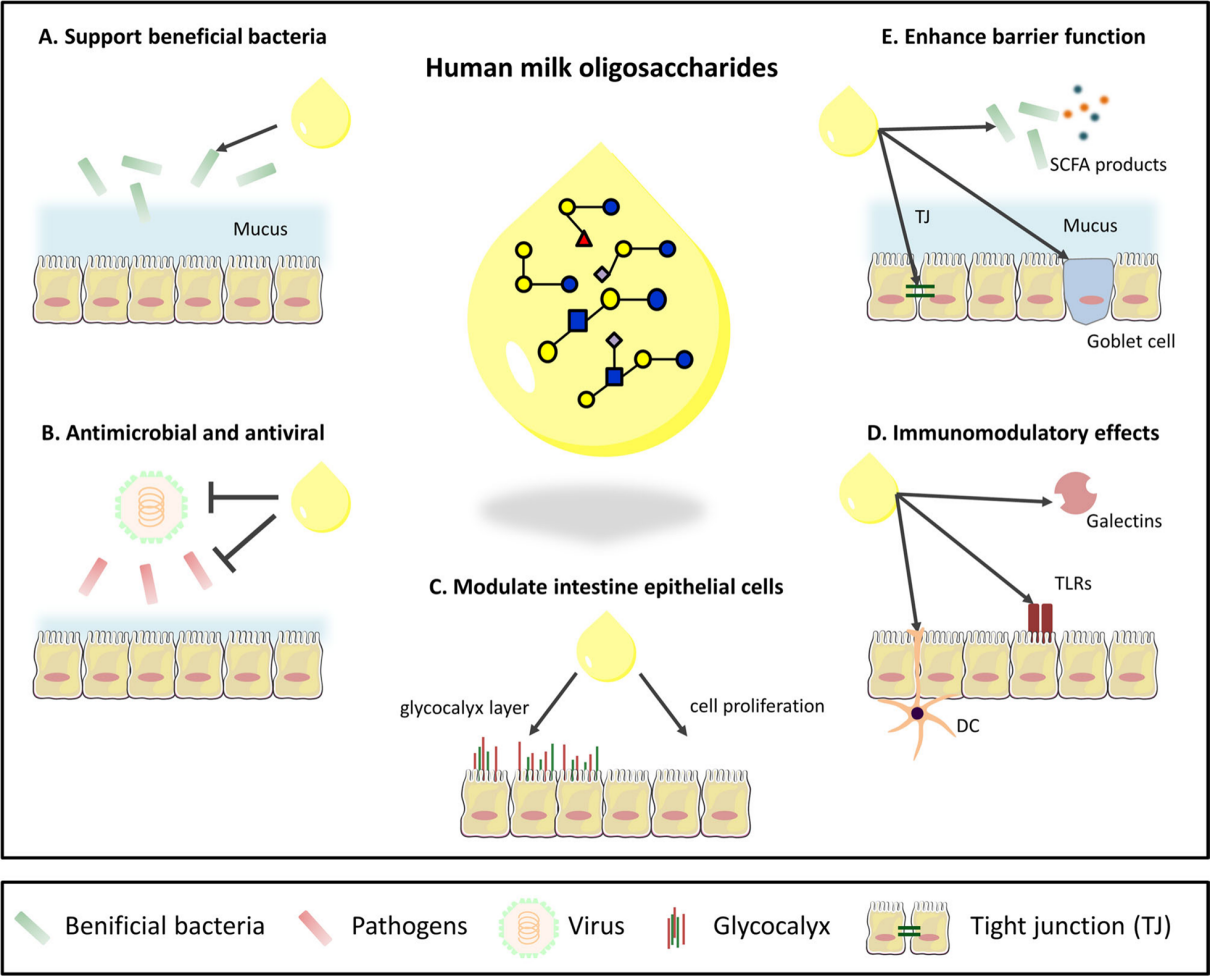
Overview of known functions of HMOs in the intestine

Human milk oligosaccharides (HMOs) abundantly present in breast milk, represent a significant component of human milk carbohydrates ranking third in terms of abundance. The average concentration is approximately 12.9 g/L in mature milk and rises to around 20.9 g/L four days postpartum. HMOs exhibit remarkable structural diversity [27, 28]. Ranging from two to thirty-two sugars in length, HMO composition differs significantly from that found in other mammals [29, 30]. Notably, HMOs consist of five distinct monosaccharides arranged in various sequences and orientations: l-caramel, d-glucose, d-galactose, n-acetylglucosamine and n-acetylneuraminic acid [31]. Breast milk contains over 200 known oligosaccharides, all of which have lactose at the reducing end [32]. Additionally, 200 breast milk oligosaccharides have been identified, with successful resolution of structures for 100 of them [33, 34]. While many believe that human milk oligosaccharides (HMOs) lack nutritional value for infants, they are actually synthetic glycosyltransferases capable of synthesizing similar structures on other human mucous membranes. Moreover, HMOs function as prebiotics by selectively promoting the growth of beneficial organisms [35]. Previously thought to be sterile, but breast milk has been found to contain microbes whose composition varies based on the mother’s characteristics and breastfeeding process [36, 37]. Acting as a prebiotic in the infant’s gut, HMOs prevent colonization by pathogenic bacteria while promoting probiotic growth (Fig. 1) [31]. Furthermore, HMOs have demonstrated efficacy in treating diarrhea and respiratory infections. For instance, one study showed that HMOs effectively inhibit Group B streptococcus GBS microbiota infection [2], prompting further investigation into their potential against ESKAPE (*Enterococcus faecium, Staphylococcus aureus, Klebsiella pneumoniae, Acinetobacter baumannii, Pseudomonas aeruginosa, and Enterobacter* species) pathogens, a group comprising *Enterococcus faecium*, *Staphylococcus aureus*, *Klebsiella pneumoniae*, *Acinetobacter baumannii*, and *Enterobacter pseudomonas aeruginosa* known for their antibiotic resistance capabilities [38–40]. The study selected staphylococcus aureus and *Acinetobacter baumannii*, both of which can cause severe health complications in infants. The findings revealed that HMO exhibited inhibitory activity against *Acinetobacter baumannii*, albeit less potent than GBS. Surprisingly, HMO had minimal impact on the growth of methicillin-resistant *Staphylococcus aureus* (MRSA) [41] (Table 2). Analysis using live/dead baclight demonstrated that increasing concentrations of HMO led to enhanced membrane permeability in GBS (Table 3), indicating strain-dependent antibacterial activity of HMOs. The investigation highlighted the significant influence of slight structural variations in HMOs on their antimicrobial efficacy (Table 4) [42–44]. Numerous studies have also indicated that branched chains of HMOs contribute to viral infection prevention [45], while it is true that chemical synthesis, enzymatic processes or fermentation can produce HMO molecules, these approaches remain challenging and costly. Therefore, further research into various aspects of HMOs is warranted [34]. For instance, it remains unclear how bacterial or viral infections affect the composition of breast milk. If such infections do alter the content composition of breast milk, does breastfeeding still remain the preferred method for infant feeding? These questions continue to perplex researchers.

**Table 2:** HMO antimicrobial and antibiofilm activity against various bacterial pathogens at 24 h [41]

	Maximum growth inhibition	Maximum biofifilm inhibition
*S. agalactiae*	89%	93%
*S.aureus*	none	60%
*A. baumannii*	11%	none

**Table 3:** Results of the live/dead BacLight Assay [160] live/dead cell ratio decrease from control, all groups growth in THB for 24 h

HMO concn (mg/mL)	20.5	10.25	5.25	2.56
*S. agalactiae* strain GB590	28%	28%	27%	33%
*S. agalactiae* strain CNCTC 10/84	28%	30%	43%	30%
*S. agalactiae* strain GB2	30%	27%	23%	54%

Control: grown in absence of HMOs without add supplement

**Table 4:** Summary of antimicrobial and antibiofilm activities of HMOs against GBS [42–44]

HMO	Avg growth reduction^b^	Avg viability reduction^b^	Avg biofilm reduction^c^	Avg growth reduction^b^	Avg viability reduction^b^	Avg biofilm reduction^c^
lactose (Lac)	3%	0%	0%	0%	2%	0%
2%ctose (Lac) ary of Antim	8%	0%	0%	9%	9%	0%
3-fucosyllactose (3-FL)	15%	0%	4%	0%	4%	0%
Difucosyllactose (DFL)	51%	17%	0%^d^	0%	11%	3%
Lacto-N-triose II (LNT II)	54%	12%	0%^d^	22%	8%	0%
3%%to-N-triose II (LNT I)	13%	0%	0%	0%	5%	10%
60%to-N-triose II (LNT I)	18%	0%	13%	0%	4%	9%
Lacto-N-tetraose (LNT)	24%	11%	0%^d^	0%	0%	28%
Lacto-N fucopentaose I (LNFP I)	1%	24%	35%	0%	10%	0%
Lacto-N-fucopentaose II (LNFP II)	31%	15%	0%	0%	9%	0%
LS-tetrasaccharide a (LST a)	38%	23%	0%^d^	42%	25%	0%^d^
Disialyllacto-N-tetraose (DSLNT)	28%	18%	0%	18%	21%	0%
Lacto-N-neotetraose (LNnT)	42%	13%	0%	5%	4%	13%
Lacto-N-fucopentaose III (LNFP III)	26%	14%	0%	0%	9%	6%
Lacto-N-neohexaose (LNnH)	48%	12%	0%^d^	39%	15%	0%
Para-lacto-N-neohexaose (para-LNnH)	23%	9%	0%	0%	8%	13%
LS-tetrasaccharide c (LST c)	15%	16%	0%	35%	18%	0%
Heterogeneous HMO extract	82%	23%	N/A	73%	24%	N/A

^a^Strongest activity is bolded. ^b^Average over 24 h of growth. ^c^Average at 24 h of growth. ^d^Significantly increased biofilm formation

### Protein

The bioactive proteins present in breast milk play a crucial role in the growth and development of infants, making them an important research focus in clinical medicine [46, 47]. Proteomics studies have identified over 400 types of proteins in human milk, with some exhibiting immune response, cell metabolism, and protein metabolism functions [48]. These proteins can be classified into three groups—whey proteins (60–80%), casein proteins (20–40%), and MFGM proteins (1–4%) based on their abundance levels [49]. Lactoferrin, lysozyme, secretory IgA (sIgA), haptocorrin (vitamin B12-binding protein), α-lactalbumin, bile salt stimulated lipase (BSSL), κ-casein and β-casein are among the bioactive components found within these groups [4]. Additionally, after β-casein brown peptide metabolism or α/β/milk protein digestion occurs along with glycan-protein binding release by lactoferrin decomposition results in numerous small molecules that exhibit various biological activities [50–53]. Current research has demonstrated opioid activity as well as antibacterial and immunomodulatory properties for many of these peptides [54, 55]. Some of the different types of proteins in breast milk and their specific biological activities have been explored. How to add these proteins, which are crucial to the growth and development of infants and young children, into infant formula is a prominent research focus among various formula manufacturers.

#### Casein

Casein, one of the three proteins in human milk, belongs to the squamous protein family that is found in all typical mammals and comprises four isoforms αs1-casein, αs2-casein, β-casein and κ-casein. While bovine milk contains αs2-casein, it is absent from human milk [56], The primary caseins in human milk are β- and κ-caseins with a low concentration of α-casein [57], where β-casein accounts for 70% of the total caseins [58]. Human β-casein has six subtypes ranging from 0 to 5 based on its degree of phosphorylation [59]. Among these caseins isoforms, κ-casein stabilizes insoluble α- and β-casein by forming colloidal suspension. However, due to the absence of disulfide bonds formation capability in caseins results in a tangled network of micelles. Furthermore, although only constituting about 13 percent of human milk protein content, but it serves as a primary source for active peptides during infant development that corresponds to their growth rate [60, 61]. Some study focused on β-casein and κ-casein, with the latter being a high protein glycosylation that acts as a bacterial adhesion inhibitor, preventing helicobacter pylori from adhering to the human intestinal wall. This explains why breastfed babies are less susceptible to helicobacter pylori infection [62], Additionally, lactoferrin also exhibits similar effects, highlighting the synergistic effect of milk proteins. The glycans in casein have a structure similar to carbohydrates found on gastrointestinal mucosal cells’ surfaces and can act as soluble bait for pathogens [63, 64]. Furthermore, after casein cleavage, carbohydrate-containing glycomacropeptide has antibacterial adhesion properties and other biological activities [65]. β-casein is unique due to its multiple phosphorylated amino acids on the backbone that facilitate calcium absorption through smaller casein phosphopeptides formation upon digestion. β-casomorphine (BCM), formed by decomposing β-casein into small molecule active opioid peptides such as sleep induction, mucosal development immune regulation antioxidant satiation gastrointestinal function [4, 66]. Moreover Casein-197, a new endogenous peptide in human milk also has an antibacterial effect mainly through inhibition of cell membrane [67]. The investigation into endogenous peptides continues to progress.

After analyzing casein activity and composition of human casein, research on casein of other mammals also have many valuable directions. Firstly, it is important to investigate the differences in levels and properties of casein between humans and bovines (Table 5). Secondly, the digestibility and kinetics of casein vary among different mammalian milks within the human intestinal tract. Most caseins are hydrolyzed by duodenal fluid during digestion. Notably, horse milk exhibits a significantly faster digestion rate compared to cattle and sheep milk. This phenomenon may be attributed to its low content of κ-casein and larger size of casein micelles, which render it more susceptible to enzymatic hydrolysis. Additionally, horse milk contains β-casein with varying degrees of phosphorylation that contribute to this rapid digestion process. Interestingly, lysozyme found in mare’s milk remains largely intact after being digested by the human body [68]. Furthermore, investigating immune and antimicrobial activities in mammalian milks is an essential research direction since some individuals experience allergic reactions when consuming cow’s milk. However, such reactions are rarely observed with donkey milk consumption due to lack of cross-reactivity between purified β-caseins from donkeys and cows [69]. Moreover, donkey milk (DM) shares certain components and functions with breast milk while also possessing unique structural features and compounds. While lactoferrin is the active component responsible for antibacterial action in human milk, DM exhibits even greater effectiveness through lysozyme, L-amino acid oxidase, and various other protein components. It is worth noting that DM possesses more stable physical and chemical properties compared to other ruminants, thereby better preserving its nutritional properties [70]. However, bovine milk also holds significant activity and research value. For instance, κ-casein found in both human and mature cow milk demonstrates anti-human rotavirus (HRV) activity. This phenomenon may be attributed to the high glycosylation of milk κ-casein and human κ-casein. Subsequent experiments revealed a notable decrease in anti-HRV activity following deglycosylation of κ-casein [71]. Currently, the analysis of protein structure and properties in human milk remains incomplete while ongoing research on mammalian milks continues.

**Table 5:** Differences in levels, properties, between caseins in human and bovine milk [161–172]

Type of milk	Level (g L − 1)			
	β-Casein	κ-Casein	αs1-Casein	αs2-Casein
Human	1.25 (0.04–4.42)	0.75 (0.1–1.72)	0.33 (0.04–1.68)	
Bovine	8.6–9.3	2.3–3.3	8.0–10.7	2.8–3.4

Type of milk	Molar mass			
	β-Casein	κ-Casein	αs1-Casein	αs2-Casein
Human	23.9–24.2	19.0	21.0	
Bovine	23.9–24.1	19.0	22.1–23.7	25.2–25.4

Type of milk	Structure			
	β-Casein	κ-Casein	αs1-Casein	αs2-Casein
Human	Multi-phosphorylation (0–5P)	High glycosylation (carbohydrate weight: 40%)	9 potential phosphorylation sites; -0P or partially phosphorylated form	
Bovine	Major -5P, occasionally -4P form	Carbohydrate weight: 10%	Major -8P and -9P form	10–13P form

#### Whey proteins

Although the proportion of whey protein in milk is relatively small. Whey protein is extensively utilized due to its rich content of bioactive substances and physiological effects. It has been widely incorporated into various whey protein beverages and food products. Whey protein exhibits hypotensive properties, with hypotensive whey peptides considered as key components responsible for this effect. Numerous bioactive peptides have been designed to inhibit angiotensin-converting enzyme (ACE). For instance, α-lactalbumin (α-La), β-lactoglobulin (β-La), and glycomacropeptide synthesized by plant enzymes demonstrate significant ACE inhibitory activity both in vitro and during simulated gastrointestinal digestion. Despite the isolation and testing of several antihypotensive whey peptides in vivo, their precise mechanism of action remains unclear [72, 73]. Whey protein exerts antibacterial effects against a variety of microorganisms including gram-negative bacteria (such as bacillus subtilis, *Escherichia coli*, and *Pseudomonas aeruginosa*), yeast, fungi, while also collaborating with lactoferrin to enhance immune function. Additionally, it possesses therapeutic and regulatory cardiovascular effects when combined with other bioactive substances [74]. The presence of whey protein in human serum suggests a potential connection between human milk and serum, indicating that maternal diet may influence the composition of milk. The predominant whey proteins found in human serum include α-whey protein, lactoferrin, lysozyme, IgA, and serum albumin [49]. β-lactoglobulin is the primary whey protein in milk but is absent in breast milk. However, any beta-lactoglobulin detected in breast milk is likely derived from the mother’s diet [75]. This suggests that the protein intake of the mother’s daily diet may influence to some extent the type and amount of protein in breast milk.

Lactoferrin is commonly added to formula as a supplement for essential nutrients required by the body. Since it cannot be directly extracted from human milk, lactoferrin must be obtained from mammalian sources, such as bovine or goat milk [76]. Although lactoferrin extracted from mammalian milk shares similar physicochemical properties with its counterpart in human milk, there might be notable differences in physiological activity. Previous studies have suggested a protective effect of lactoferrin against infections in preterm infants [77]. However, recent large-scale trials have not demonstrated a clear association between lactoferrin supplementation and reduced mortality, morbidity rates or infection risk among preterm infants.

Lysozyme present in human milk is an acid-resistant glycoprotein with a molecular weight of approximately 15 kDa. It possesses antimicrobial properties against gram-positive bacteria by attacking their cell walls and can also synergize with lactoferrin to combat gram-negative bacteria. Lysozyme has been shown to reduce diarrhea duration following acute diarrhea episodes among children after rehydration therapy [78]. Furthermore, lysozyme exhibits inhibitory effects on HIV replication [49]. Although this inhibition only applies to free viruses, it provides insights into addressing the severe global issue of HIV transmission.

The most effective whey protein for inflammation and viral infections is sIgA immunoglobulin, which possesses the ability to recognize receptor binding sites and directly impact bacterial virulence. This capacity to suppress the immune response against microbes is referred to as immune rejection [79]. T immune rejection of sIgA can be triggered by bacteria as well as potential allergenic antigens, highlighting its significance in the human immune system. SIgA and immunoglobulin G have been observed to form complexes with antigens present in breast milk [80]. The recent COVID-19 outbreak has demonstrated that sIgA exhibits immunity towards it, eliciting an immune response when present in breast milk [12]. These findings also suggest that sIgA may serve as a natural antibody against various pathogenic agents. However, further research is required to elucidate its mechanism and scope of action. Other whey proteins are currently under exploration, with some garnering significant attention and research due to their specific functions and relatively high concentrations. Notable examples include a vitamin B12 binding protein and osteopontin, both exhibiting potential antibacterial activity.

#### Milk fat globule membrane proteins

Although the protein content of MFGM in milk is only approximately 1%, it encompasses over 100 proteins that are unique to human milk. The primary molecular functions attributed to MFGM proteins include guanine nucleotide binding and lipoprotein binding, which play crucial roles in lipid synthesis and secretion. Many of these bioactive proteins have been found to possess antibacterial and antiviral activities [81–83]. However, the specific mechanisms and applications of them, are still being investigated.

### Phenols

In a search for references on key nutrients in human milk, it was observed that the majority of studies or reviews overlooked an important class of compounds known as phenols.

Phenols have been extensively studied and shown to possess potent biological activities. Furthermore, it has been demonstrated that the composition of maternal milk during lactation is influenced by various bioactive substances present in the mother’s daily diet, which enter the milk through plasma and are subsequently consumed by the infant [84]. Notably, isoflavones, a common phenolic substance found in human diets and belonging to flavonoids, were discovered to exist in breast milk primarily as glucuronic acid and sulfate conjugates based on numerous experiments [85]. Various sulfuric acid conjugates along with certain glucuronic acids (such as 4-hydroxybenzoate glucuronic acid, 3-hydroxybenzoate glucuronic acid, dihydroferulic acid glucuronide) and some free phenolic acids (vanillic acid, iso-vanillic acid) were detected both in urine and breast milk samples. Initially believed to be only excreted via urine when infants were fed soy formula exclusively [86]. Subsequent experiments revealed that consumption of soybeans or soy products by lactating mothers significantly increased isoflavone content in breast milk [87]. Moreover, prolonged maternal intake of soy-based foods resulted in more consistent and average levels of isoflavones within breast milk samples [88]. Additionally noteworthy is the higher bioavailability of isoflavones observed among infants and young children compared to adults, even infants as young as 4–6 months old exhibited efficient intestinal absorption of these compounds [89]. The high prevalence of soy consumption in certain Asian countries may contribute to elevated levels of isoflavones among Asian children. The low incidence of breast and prostate cancer in these populations may be attributed to their high exposure to isoflavones [90]. Furthermore, all samples contained significant amounts of important phenols such as hesperidin, naringin, and quercetin. Quercetin Q concentrations were found to be time-dependent, with no significant decrease observed in breast milk during the period when quercetin Q was tested. This suggests that this compound may accumulate in the body even when dietary intake reduced. Flavan-3-ols (epicatechin, epicatechin gallate, epigallocatechin gallate), flavonols (quercetin and kaempferol) and flavanones (naringin and hesperetin) were detected at the highest concentrations among the various phenolic compounds analyzed [91]. Citrus fruits and juices, such as oranges and grapefruits, are particularly rich sources of flavonoids. Additionally, gallic catechin and gallic acid ester content were found to be high in the experimental testing table but only present in eight individuals. Meanwhile, breastfed babies metabolize epicatechin through host-microbial interactions [92]. Breastfeeding mothers are typically advised to avoid consuming foods or drinks containing flavanol-3-ols due to their caffeine content. However, tea consumption cannot always be completely avoided despite limitations on its intake. Another study reported increased levels of polyphenols in cranberries, as well as elevated concentrations of polyphenol metabolites in plasma, urine and breast milk [93]. These findings are consistent with the presence of previously identified phenolic metabolites such as parental anthocyanins, uroliths, benzoic acid derivatives and cinnamic acid derivatives.

In addition to the well-established antioxidant properties of phenolic compounds, certain flavonoids also exhibit promising anti-cancer potential. Moreover, with regard to the growth and development of infants and young children, phenolic compounds demonstrate evident neuroprotective and antibacterial effects [94]. Such as caffeic acid, exhibit inhibitory effects on the growth of bacteria such as escherichia coli, staphylococcus aureus, bacillus cereus, listeria monocytogenes and certain yeasts [95]. Caffeic acid demonstrates significant antioxidant and antibacterial activities in cosmetic emulsions with an acidic pH range of 3–5. Moreover, it has been observed that caffeic acid exerts its antimicrobial influence on various microorganisms (escherichia coli, pseudomonas aeruginosa, bacillus wax-like species, thermophilic root kokuria species, hyperplastic Staphylococcus aureus strains, monocyte Listeria species and Candida albicans) through aromatic and phenolic compounds by affecting the cytoplasmic membrane structure and function [96]. This leads to alterations in transport activity and cellular membrane reactions resulting in increased permeability of the cell membrane as well as loss of cellular components.

Therefore, lactating mothers can appropriately increase the consumption of flavonoid and other phenol-rich foods, thereby facilitating nerve growth and development in infants, reducing the prevalence of major diseases, and enhancing the immune system development in lactating infants. The presence of various phenolic compounds in breast milk is crucial for the growth and development of infants and young children. This aspect has become a focal point in guiding infant nutrition research as well as one of the key areas of investigation for formula milk powder.

### Cytokines

In the growth and development of the human body, a diverse array of cytokines plays crucial roles. During infancy, these cytokines appear to be more prominent as they can induce or inhibit inflammation, facilitate cell communication, modulate cognitive function and participate in stem cell differentiation. Cytokines are primarily produced by immune cells in the human body and exhibit variations in their structural sizes. Cytokines encompass various types including chemokines, adipokines, interferons, interleukins, transforming growth factor (TGF), and tumor necrosis factor (TNF) [97–99]. Chemokines specifically contribute to blood vessel growth, embryo development, and organogenesis while also promoting inflammation and playing a significant role in disease pathogenesis. Notably, they have been closely associated with autoimmune diseases and tumorigenesis within the human body—highlighting their importance as research subjects. Interferons represent cellular factors rapidly produced by the host upon viral infection with particular relevance to double-stranded RNA viruses. Interleukins are another class of immune-related cytokines that primarily function as signal transducers within the immune system [100, 101]. Adipocytokines encompass a range of compounds, including leptin, adiponectin, resistin, visfatin, chemokine, angiotensin and other molecules. The primary function of adipokines is to directly or indirectly participate in apoptosis, angiogenesis, atherosclerosis and inflammation. Furthermore, it has been established that adipokines also exhibit regulatory effects on human blood pressure [102, 103]. Tumor necrosis factor α (TNF-α) is a cytokine consisting of 157 amino acids and exists in two forms as pro-inflammatory molecules. It possesses diverse biological activities such as tumor cell killing or inhibition, enhancement of neutrophil phagocytic ability, anti-infection properties, endogenous pyrogenic effects, facilitation of myeloid leukemia cell differentiation into macrophages and promotion of cell proliferation and differentiation [104–106]. The diverse array of cytokines plays pivotal roles in the human body and possesses significant research and utilization value.

In addition to the classification analysis of cytokines, various studies have focused on the biological activities exhibited by cytokines present in breast milk. Cytokines found in breast milk are crucial for neonatal development. Breast milk serves as the main source of cytokines during infancy with particular emphasis on anti-inflammatory factors. These factors demonstrate potent anti-inflammatory activity which can modulate inflammatory processes, stimulate wound healing and activate maintain immune responses within the body. For instance, TGF-b regulates cellular homeostasis and inflammation while inducing or inhibiting immune responses. Animal experiments have demonstrated that TGF-b exerts protective effects by reducing intestinal cell apoptosis [107]. In a study, chondroitin sulfate proteoglycan was found to isolate TGF factors in breast milk. After enzymatic decomposition, the protective, growth and anti-inflammatory effects of TGF2 were detected [108]. However, TGF-like cytokines were positively correlated with TH1, TH2 cells as well as other allergic factors. This correlation has led to the deliberate absence or reduction of TGF-like cytokines in certain formula milk powders to minimize the risk of allergies [109]. Another cytokine called MFG-E8 has been experimentally proven to significantly increase mortality rates in mice when lacking this particular cytokine. Neonatal inflammation and sepsis-related mortality are particularly affected by its absence. These findings suggest that LGF8 may hold potential as a therapeutic agent for neonatal leukemia treatment [110]. Additionally, another experiment demonstrated that this cytokine plays a significant role in the pathogenesis of rheumatoid arthritis, subsequent bone loss exacerbating symptoms and prevalence of arthritis. Therefore, targeting this specific cytokine could be considered for therapeutic interventions against arthritis [111–113]. After a comprehensive understanding of cytokines, an intriguing phenomenon emerges. The uncontrolled release of a large quantity of pro-inflammatory cytokines by the immune system leads to the occurrence of a cytokine storm, characterized by an excessive and unregulated immune response that triggers cascading reactions an unstoppable process. It is well-established that cytokines in the human body are subject to both positive and negative feedback regulation, maintaining the delicate balance between growth, development as well as inflammation production, encompassing both detrimental and beneficial effects on human health. However, the precise origin of cytokines in breast milk remains elusive. One potential source could be mammary epithelial cells. Additionally, white blood cells such as neutrophils, monocytes, macrophages and lymphocytes migrate to the breasts through lymphatic and systemic circulation [114–118]. Nevertheless, whether maternal inflammation-induced cytokine storms can inflict similar harm on infants necessitates further investigation.

## Research and development prospect of breast milk lipids

### Lipids in breast milk

Lipids are essential components in all types of milk, including human milk, where they play a crucial role. They can be broadly categorized into triacylglycerols, phospholipids, cholesterol and free fatty acids [5]. Fatty acids encompass both saturated and unsaturated forms. The addition of unsaturated fatty acids to formula is often found in various media and advertisements due to their significant impact on neonatal health [119]. Consequently, numerous studies have focused on investigating the content and types of lipids present in breast milk to provide guidance for infant formula development. Experimental findings indicate that triglycerides account for approximately 98.7% of general breast milk lipid content, while cholesterol represents about 0.3%, free fatty acids around 0.1%, phospholipids roughly 0.9%, with trace amounts of other lipid contents detected as 1,2-diacylglycerol [120]. Most of these lipids exist within human lactolipid microspheres, particularly those with notable biological activity such as sphingomyelin, phosphatidylcholine, phosphatidylinositol, phosphatidylserine and phosphatidylethanolamine. These microspheres also contain short-chain or medium-chain fatty acids esterified at position 3 of the glycerol skeleton. However, palmitic acid is predominantly esterified at position 2 in human milk [121]. The unique structure of long-chain palmitic acid provides effective protection against saponification with calcium and magnesium, thereby preventing its hydrolysis into free palmitic acid. Infants struggle to digest and absorb free fatty acids, resulting in impaired absorption of essential minerals like calcium [122]. Research has indicated that the presence of free palmitic acid can also contribute to infant constipation [123], which explains why some infants experience relief from constipation and dry stool upon discontinuing formula milk powder consumption in favor of breast milk.

### Lipids in milk fat globule membrane

**Fig. 2:**
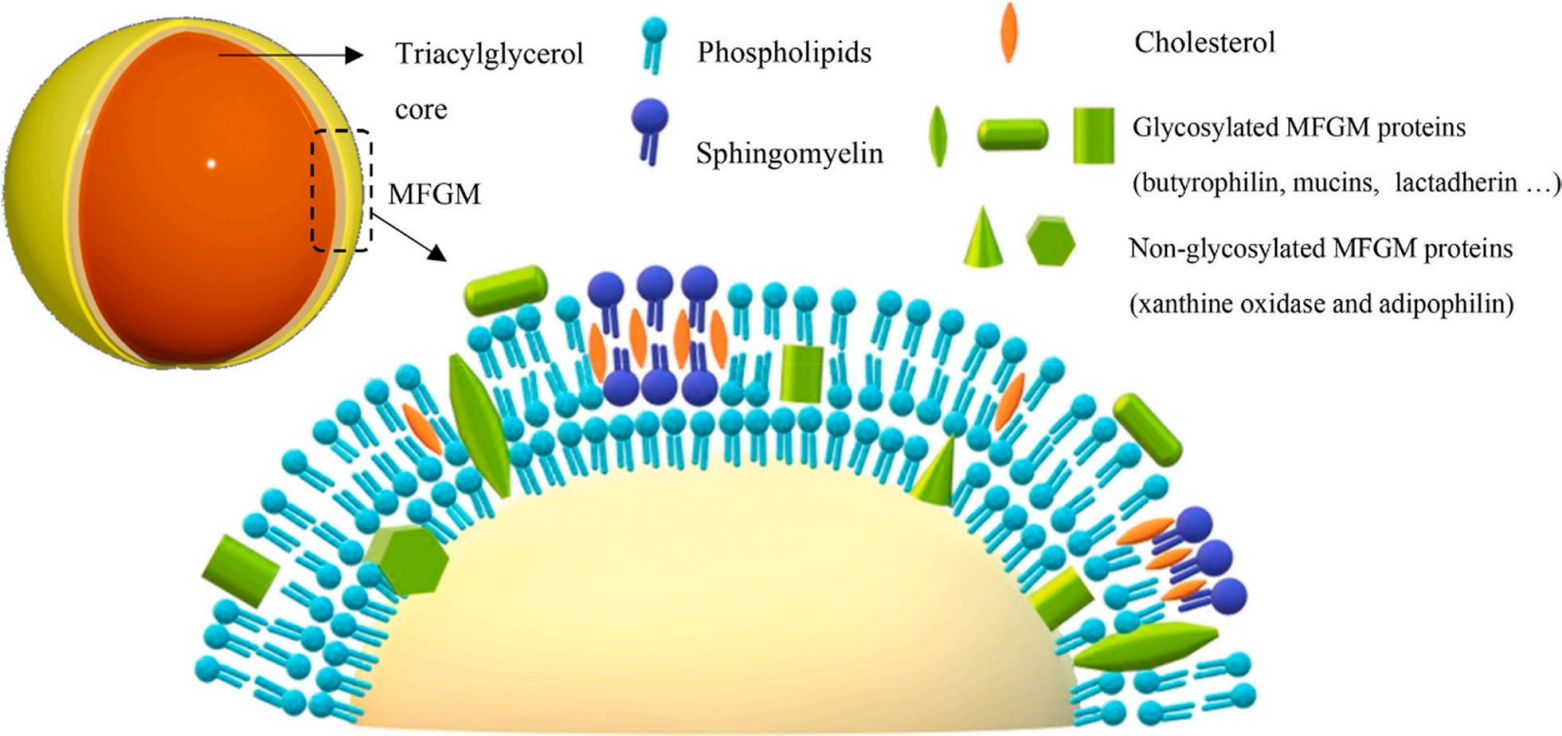
Human milk fat globules and human milk fat globule membrane

MFG represents the primary form of lipids in human and other mammalian milk, with a highly similar structure between mammals and humans. The core of MFG is TG, which is packaged within MFGM a three-layer membrane structure composed of phospholipids, cholesterol, and MFGM protein (Fig. 2). The inner layer of this lactoglobular membrane’s three-layered structure consists of a monolayer formed by the endoplasmic reticulum of breast cells while the outer bilayer comprises a bilayer formed by the apical membrane of breast epithelial cells. Due to its complex composition primarily consisting of lipid, cholesterol and milk lipid globular membrane protein components, MFGM contains various bioactive substances that contribute to milk’s bioactivity [83, 124]. In terms of lipids, the lipids in milk can be broadly categorized as polar and nonpolar lipids. The polar lipids in milk are primarily found on the milk lipid globule membrane, with lactomyelin (glycerophosphingomyelin and sphingomyelin) serving as representative polar lipids along with other types of lipids such as phosphatidylcholine (PC), phosphatidylethanolamine (PE), phosphatidylinositol (PI) and phosphatidylserine (PS). These lipids are commonly classified and reported as phospholipids due to their significant presence in milk. Given that lecithin has been shown to promote infant brain development [125], it has garnered increasing attention and is now used as a reference standard for fat content in formula milk powder [126]. Lipids in MFGM exhibit a multitude of significant biological activities. For instance, phospholipids play a crucial role in mediating cell growth and differentiation [127], enhancing neural and cognitive development [128, 129], improving brain function and neural plasticity [130], promoting postpartum neuromuscular development [131], regulating postprandial cholesterol levels and exerting anti-infective effects [132–134]. Sphingomyelin has potential anticancer properties [135], phosphatidylcholine possesses antitoxin properties [136] and the hydrolysate of phospholipids demonstrates antibacterial activity [137]. Recent experiments have provided evidence for statistically significant anti-inflammatory effects of MFGM lipids [138], as well as their inhibitory and attenuating effects on Escherichia coli-induced diarrhea [139]. Furthermore, they may also contribute to the inhibition of rotavirus infection [140]. The diverse range of biological activities exhibited by simple lipids present in MFGM underscores the need for further comprehensive research on this subject. Simultaneously, existing studies serve as valuable references providing important insights into infant formula research.

### Lipids in breast milk during different periods

Breast milk serves as the primary source of nutrition for infant growth and development. Extensive research conducted by numerous scholars has revealed variations in lipid composition and content among breast milk secreted by mothers during different lactation periods and delivery cycles. For instance, from 1 to 6 months postpartum, there is a general decline in overall lipid content. Older mothers exhibit significantly lower levels of polyunsaturated fatty acids (PUFAs) and linoleic acid compared to younger mothers, while proportions of eicosapentaenoic acid, docosahexaenoic acid (DHA), monounsaturated fatty acids, n-3 LC PUFAs, n-3 and n-6 are higher in older mothers [141]. However, experimental evidence does not conclusively establish a direct correlation between fat content and age. Among pre-pregnancy women with higher body mass index values, the concentration of monounsaturated fatty acids tends to be lower. Moreover, an increase in body mass index corresponds to elevated energy, fat, protein and galactose levels. Nevertheless, these differences were found to be statistically insignificant after conducting several experiments [142]. Preterm breast milk exhibits significantly higher average fat values than full-term breast milk does. Moreover, average fat content demonstrates an upward trend with decreasing gestational weeks [25]. The concentration of phospholipids (PL) in preterm milk exhibits a significant decrease, while maintaining stability from colostrum to maturity. Conversely, the concentration of phosphatidylcholine (PC) in colostrum is initially higher during the transition and mature stages, gradually declining to a lower level. In comparison to full-term milk, preterm milk demonstrates an elevated presence of medium chain fatty acids and higher levels of phosphatidylethanolamine (PE) and PC [143]. Furthermore, the content of 18:1 N-9 fatty acids in preterm milk surpasses that found in full-term milk. Although no notable difference exists regarding the content of 18:1 N-9 in full-term milk, while there is a discernible downward trend observed within preterm milk. Additionally, another N-9 fatty acid known as 20:1 N-9 displays significantly higher levels in term milk compared to those present in preterm infants [126]. The arachidonic acid (20:0) content is lower in preterm infants’ milk, while the monounsaturated fatty acid (MUFA) content is significantly higher in term milk compared to preterm infants’ milk. Additionally, the alpha-linolenic acid (ALA) content is higher in preterm infants’ breast milk than in full-term breast milk [144]. Furthermore, variations exist in the levels of 235 different lipids between preterm and full-term breast milk samples [145]. However, studies have demonstrated that there are no differences in the proportions of long-chain polyunsaturated fatty acids (LCPUFA) between full-term and preterm milk samples [146], as well as no disparities in the ratios of arachidonic acid (ARA) and docosahexaenoic acid (DHA) between full-term and preterm milk samples one week post-production [147]. Some experiments indicate that within the first week after delivery, ARA and DHA contents are nearly twice as high in preterm infants compared to full-term milk [148]. Reported higher levels of ARA in breast milk from full-term mothers compared to preterm samples. Observed a gradual decline in the proportion of ARA and docosahexaenoic acid (DHA), particularly in full-term milk samples, as the levels of ARA and DHA increased by 1.5–2 times in preterm milk during the sixth month of lactation [149]. This experiment highlights that breastfeeding mothers of preterm infants are unable to compensate for the increased demand for long-chain polyunsaturated fatty acids (LCPUFA) resulting from premature birth. Therefore, solely considering changes in lipid composition within breast milk allows us to draw a conclusion that this paper aims to provide a reference for formula milk powder by summarizing and observing relevant characteristics of breast milk. However, there remain numerous unresolved issues regarding the study on composition and changes in breast milk, along with several new directions worthy of further investigation.

### Lipids in mammalian milk provide a reference for formula

Breast milk is the optimal source of nutrition for infants during early stages of growth and development. However, when breastfeeding is not possible, infant formula serves as a suitable alternative. Typically, formula milk powder consists of artificially extracted materials or plant extracts [150, 151]. Nevertheless, current research reveals several limitations associated with this method of formula production. Consequently, an increasing number of formula milk powders are incorporating milk from different mammals to bridge the gap between artificial formulas and breast milk. Notably, variations in fatty acid composition exist among different mammalian milks [152]. Goat milk is believed to be more easily digestible than cow’s milk due to its resemblance to the nutritional profile required for infant growth and development [153–155]. Its uniqueness lies in having up to 70% of palmitic acid located at the sn-2 position within triglyceride molecules [121], facilitating easier digestion of fatty acids by infants while reducing calcium loss. In contrast, most palmitic acid in cow’s milk is situated at the sn-1, -3 positions within triglyceride molecules. Both goat milk and cow’s milk serve as natural sources of sn-2 palmitic acid [156]. Studies have demonstrated that goat’s milk exhibits a higher fat content of 6.9 ± 1% compared to well-known mammals [157]. Conversely, mare’s milk (1.21%), human milk (3.64%), and cow’s milk (3.61%) display lower fat contents, while certain other mammals such as hat seal (61%), reindeer’s milk (22.46%) exhibit significantly higher fat contents [158] and water buffalo milk reaching up to 15%. However, the elevated cholesterol levels in goat’s milk exert a protective effect on infants and facilitate cholesterol metabolism during later stages of infancy. Ruminant and human milks demonstrate considerably lower concentrations of free fatty acids and phospholipids compared to mare’s and donkey milks. Non-ruminant milks exhibit low proportions of saturated fatty acids and monounsaturated fatty acids but high levels of unsaturated fatty acids, whereas goat’s milk displays the highest concentration of short-chain fatty acids among all studied species [159]. Based on these findings along with the known effects of fatty acids on the human body, the control of lipid and other nutrients in formula milk powder must be strengthened to mimic the composition of human breastmilk lipids while also incorporating polar lipids as long-chain polyunsaturated fatty acid sources in infant formulas, and reference to the composition of other mammalian milk to further optimize infant formula to meet the nutritional needs of infants in various situations. Furthermore, regulating the composition and content of phospholipids in powdered formula based on those found in human breastmilk MFGM is essential for meeting infants’ varying needs throughout different developmental stages.

## Conclusion

An increasing number of studies are being conducted on breast milk, primarily focusing on the identification and exploration of various bioactive components present in breast milk, with the aim of discovering potential therapeutic precursors for certain diseases. Alternatively, through comprehensive analysis and characterization of the composition and content of diverse substances and bioactive compounds in breast milk, along with integration of experimental data and findings, it is recognized by the World Health Organization as a crucial source of nutrition during infant growth and development. Consequently, these data can serve as an accurate reference for developing suitable alternatives to breastfeeding for infants who are unable to do so. Additionally, there exist intriguing aspects that have received limited attention thus far for instance, certain mammals lack visual observation abilities at birth but possess innate recognition capabilities towards their mothers without any visual cues while solely relying on consuming breast milk. This phenomenon prompts contemplation regarding the presence of active signaling molecules within breast milk. Irrespective of the perspective considered, research pertaining to breast milk remains a highly significant field with substantial practical implications that necessitates meticulous exploration.

## Data Availability

All the data and materials provided in the manuscript are obtained from included references and available upon request.
